# Prognostic value of olfactory nerve damage measured with thallium-based olfactory imaging in patients with idiopathic olfactory dysfunction

**DOI:** 10.1038/s41598-017-03894-4

**Published:** 2017-06-15

**Authors:** Hideaki Shiga, Junichi Taki, Koichi Okuda, Naoto Watanabe, Hisao Tonami, Hideaki Nakagawa, Seigo Kinuya, Takaki Miwa

**Affiliations:** 1 Department of Otorhinolaryngology, Kanazawa Medical University, Ishikawa, Japan; 2 Department of Nuclear Medicine, Graduate School of Medical Science, Kanazawa University, Ishikawa, Japan; 3 Physics, Kanazawa Medical University, Ishikawa, Japan; 4 Department of Diagnostic and Therapeutic Radiology, Kanazawa Medical University, Ishikawa, Japan; 5 Medical Research Institute, Kanazawa Medical University, Ishikawa, Japan

## Abstract

Idiopathic olfactory disorder is resistant to treatment, and the recovery time is long. This study investigated the prognostic value of the migration of nasally administered thallium-201 to the olfactory bulb (thallium migration to the OB), a measure of olfactory nerve damage, in patients with idiopathic olfactory disorders. Twenty-four patients with idiopathic olfactory disorders were enrolled in the study (7 women and 17 men; aged 23–73 years). We retrospectively analyzed potential prognostic markers in subjects who underwent thallium-based olfactory imaging with the nasal administration of thallium-201 before conventional treatment with the Japanese herbal medicine tokishakuyakusan and compared those data with the prognosis. Log-rank tests were performed to assess the relationship between thallium migration to the OB (<4.6% [low] vs. ≥4.6% [high]; data dichotomized at the optimal cutoff value) and the duration until recovery of the odor recognition threshold determined by a standard olfactory function test (T&T olfactometry) after the treatment. Upon statistical analysis, we found that high thallium migration to the OB was significantly correlated with better prognosis in patients. Our results suggest that patients with intact olfactory nerve fibers could be selected using thallium-based imaging for the long-term follow-up of olfactory dysfunction.

## Introduction

The most common causes of non-sinonasal-related olfactory dysfunction are postinfectious, posttraumatic and idiopathic^1^. According to previous studies, the prognosis of olfactory dysfunction due to respiratory infection, head trauma, or chronic sinusitis primarily depends on residual function and, secondarily, on gender, smoking habit, age, and parosmia^2^. Olfactory bulb volume has been shown to be a predictor of olfactory recovery in patients with postinfectious and posttraumatic olfactory loss^3^. By contrast, there are currently no accepted prognostic markers that can guide the selection and treatment of patients with idiopathic olfactory dysfunction.

The radioisotope thallium-201 (^201^Tl) is transported within the olfactory neural tract after nasal administration in rodents^4^, and this transport is markedly decreased by the transection of olfactory nerve fibers in mice^5^. Thallium has been shown to readily substitute for potassium at the (Na+/K+)-membrane ATPase activation sites^6^, and nasally administered ^201^Tl is thought to be transported into the olfactory nerve cells as a substitute for potassium.

Nasally administered ^201^Tl migrates to the olfactory bulb 24 h after ^201^Tl administration in healthy volunteers, as assessed by a combination of single-photon emission computed tomography (SPECT), X-ray computed tomography (CT), and magnetic resonance imaging (MRI)^7^. The thallium-based olfactory imaging method is called “olfacto-scintigraphy.” Nasal ^201^Tl migration to the olfactory bulb is reduced in patients with impaired olfaction due to head trauma, upper respiratory tract infection, or chronic rhinosinusitis, which are the major causes of olfactory dysfunction, compared with ^201^Tl migration in healthy volunteers^8^. The nasal administration of ^201^Tl is adapted for the visual diagnosis of olfactory nerve damage in patients with olfactory disorders. Furthermore, the biological safety of nasal ^201^Tl administration was shown in a pre-clinical study^9^.

The traditional Japanese herbal medicine tokishakuyakusan rapidly promotes the expression of nerve growth factor in the olfactory bulb and rescues neurons from damage *in vivo* ^10^. The usefulness of tokishakuyakusan has been shown for the treatment of olfactory-impaired patients with Alzheimer’s-type dementia in Japan^11^. In this study, subjects with idiopathic olfactory dysfunction received conventional treatment via the oral intake of tokishakuyakusan (TJ-23, Tsumura, Tokyo, Japan) at the standard dose of 7.5 mg per day.

Here, we retrospectively assessed the prognostic value of nasal ^201^Tl migration to the olfactory bulb (a measure of olfactory nerve damage), gender, age, odor recognition threshold determined using T&T olfactometry (T&T odor recognition threshold), smoking habit, parosmia, and olfactory bulb volume in patients with idiopathic olfactory dysfunction.

## Results

### Univariate analysis of prognostic factors in patients

To determine whether nasal ^201^Tl migration to the olfactory bulb can be used to predict the outcome of treatment in patients with olfactory disorder, we retrospectively compared the duration until recovery between patients with high (≥4.6%) nasal ^201^Tl migration to the olfactory bulb and those with low (<4.6%) nasal ^201^Tl migration to the olfactory bulb based on medical chart review. The data of nasal ^201^Tl migration to the olfactory bulb were dichotomized at the optimal cutoff value as shown in the methods.

In univariate analysis, the duration until recovery was significantly shorter in patients with high nasal ^201^Tl migration to the olfactory bulb than in those with low nasal ^201^Tl migration to the olfactory bulb (Table 1; P = 0.0003, N = 24). The recovery rate was 67% in patients with high nasal ^201^Tl migration to the olfactory bulb one year after treatment. By contrast, none of the patients with low nasal ^201^Tl migration to the olfactory bulb showed recovery at one year after treatment. The improvement in the T&T odor recognition threshold was judged according to the criteria of the Japanese Rhinologic Society^12^ (cure, T&T odor recognition threshold ≤2.0; remission, decrease in T&T odor recognition threshold ≥1.0; progress, increase in T&T odor recognition threshold of ≥1.0; stable, all other cases). Cure or remission was defined as “recovery.”

**Table 1:** Relationship between various factors and time to recovery.

Prognostic factor	12-month recovery rate (%)	Odds ratio	P value
		(95% CI of ratio)	(Log-rank test)
Thallium-201 olfactory transport rate*			0.0003
<4.6% [low]	0	1.0	
≥4.6% [high]	67	18.9 (3.9–91.2)	
Gender			0.472
male	30	1.0	
female	31	0.5 (0.1–2.8)	
Age			0.994
≥60 years	40	1.0	
<60 years	18	1.0 (0.2–4.5)	
T&T odor recognition threshold			0.055
≥5.6 [anosmia]	8	1.0	
<5.6 [dysosmia]	50	4.9 (1.0–24.4)	
Smoking habit			0.243
smokers and ex-smokers	17	1.0	
non-smokers	33	0.4 (0.1–2.0)	
Parosmia			0.658
present	17	1.0	
absent	43	1.4 (0.3–7.0)	
Olfactory bulb volume			0.577
<40 mm^3^	18	1.0	
≥40 mm^3^	50	1.6 (0.3–7.9)	

*Data dichotomized at the optimal cutoff value; the improvement in the T&T odor recognition threshold was judged according to the criteria of the Japanese Rhinologic Society [12] (cure, T&T odor recognition threshold ≤2.0; remission, decrease in the T&T odor recognition threshold ≥1.0; progress, increase in the T&T odor recognition threshold of ≥1.0; stable, all other cases). Cure or remission was defined as “recovery”.

**Figure 1:**
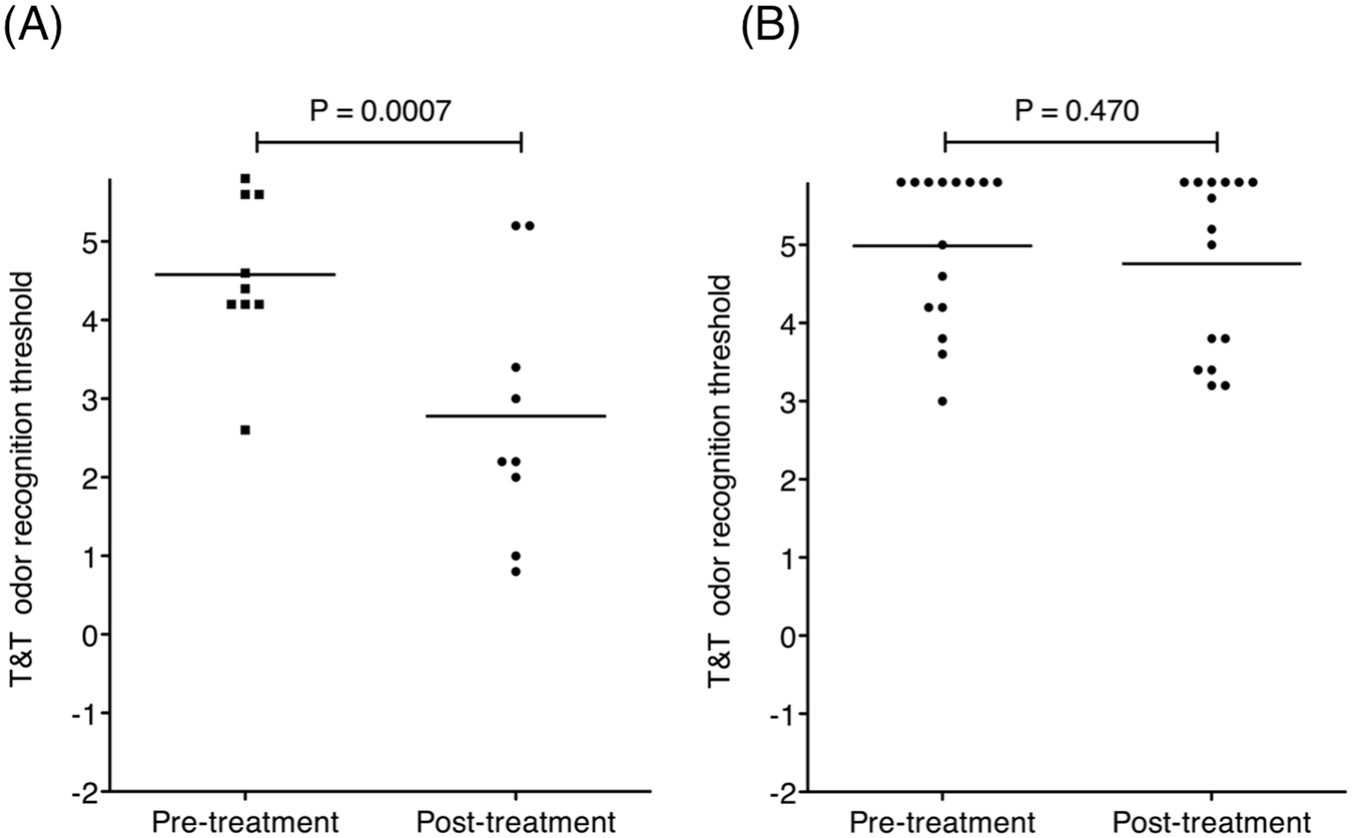
Differences in the odor recognition thresholds determined by T&T olfactometry (T&T odor recognition threshold) following conventional treatment with the Japanese herbal medicine tokishakuyakusan. Smaller numbers of odor recognition thresholds indicate higher sensitivities to odors. (**A**) Patients with high nasal ^201^Tl migration to the olfactory bulb (N = 9). (**B**) Patients with low nasal ^201^Tl migration to the olfactory bulb (N = 15). Bars indicate mean.

To assess whether differences in the T&T odor recognition thresholds were significant in the treatment of the subjects, we compared T&T odor recognition thresholds between pre-treatment and post-treatment with Mann–Whitney tests. T&T odor recognition thresholds were significantly reduced after treatment in the patients with a high Tl-201 olfactory transport rate (Fig. 1A, N = 9, P = 0.0007). By contrast, T&T odor recognition thresholds were not significantly different after treatment in the patients with a low Tl-201 olfactory transport rate (Fig. 1B, N = 15, P = 0.470). The observation time was not significantly different between patients with a high Tl-201 olfactory transport rate and those with a low Tl-201 olfactory transport rate (P = 0.152; high Tl-201 olfactory transport rate group, 8.1 ± 7.6 months [mean ± SD]; low Tl-201 olfactory transport rate group, 13.4 ± 13.8 months [mean ± SD]) (Mann–Whitney tests were used to compare the two groups).

None of the other potential prognostic factors tested (gender, age, T&T odor recognition threshold, smoking habit, parosmia, or olfactory bulb volume) was significantly correlated with prognosis in the patients with olfactory dysfunction (Table 1).

### Correlation between nasal ^201^Tl migration to the olfactory bulb and change in the T&T odor recognition threshold after treatment

**Figure 2:**
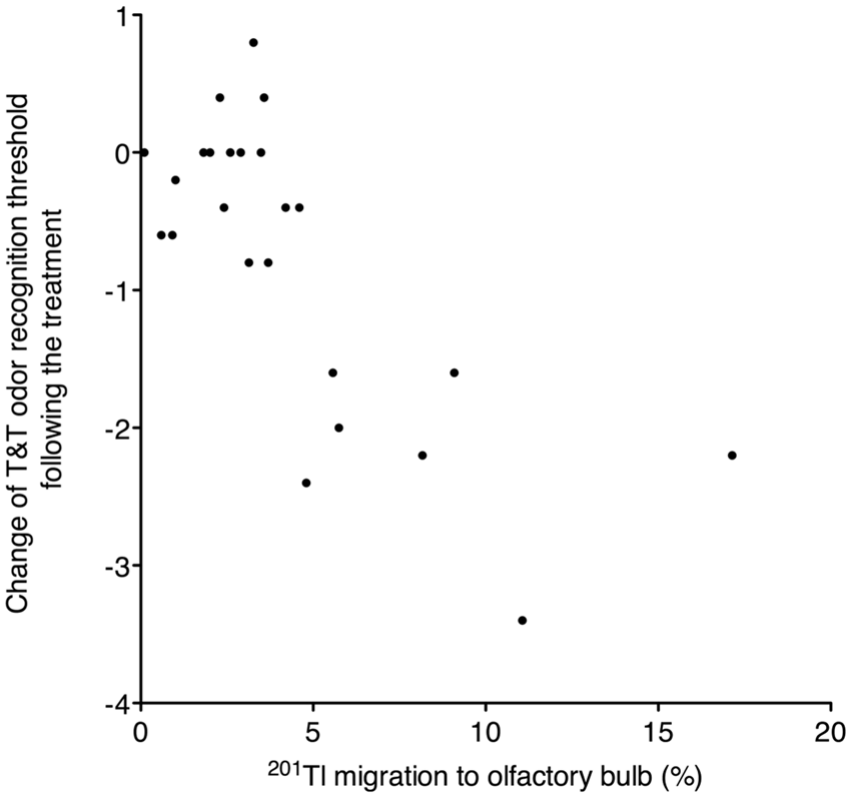
The correlation between the initial nasal ^201^Tl migration to the olfactory bulb before treatment and the change in the T&T odor recognition threshold after treatment. The nasal ^201^Tl migration to the olfactory bulb was significantly correlated with the change in the T&T odor recognition threshold following treatment (Spearman r = −0.637, P = 0.0008).

Nasal ^201^Tl migration to the olfactory bulb was significantly correlated with a change in the T&T odor recognition threshold after treatment (Fig. 2, Spearman r = −0.637, P = 0.0008).

### Correlation between nasal ^201^Tl migration to the olfactory bulb and other prognostic factors

Nasal ^201^Tl migration to the olfactory bulb was not significantly correlated with T&T odor recognition thresholds (Spearman r = −0.374, P = 0.072), age (Spearman r = 0.392, P = 0.058) or olfactory bulb volume (Spearman r = 0.205, P = 0.337). Nasal ^201^Tl migration to the olfactory bulb was not significantly different between patient groups dichotomized by gender (P = 0.341), parosmia (P = 0.271), or smoking habit (P = 0.611) (Mann–Whitney tests were used to compare two groups).

### Representative cases

**Figure 3:**
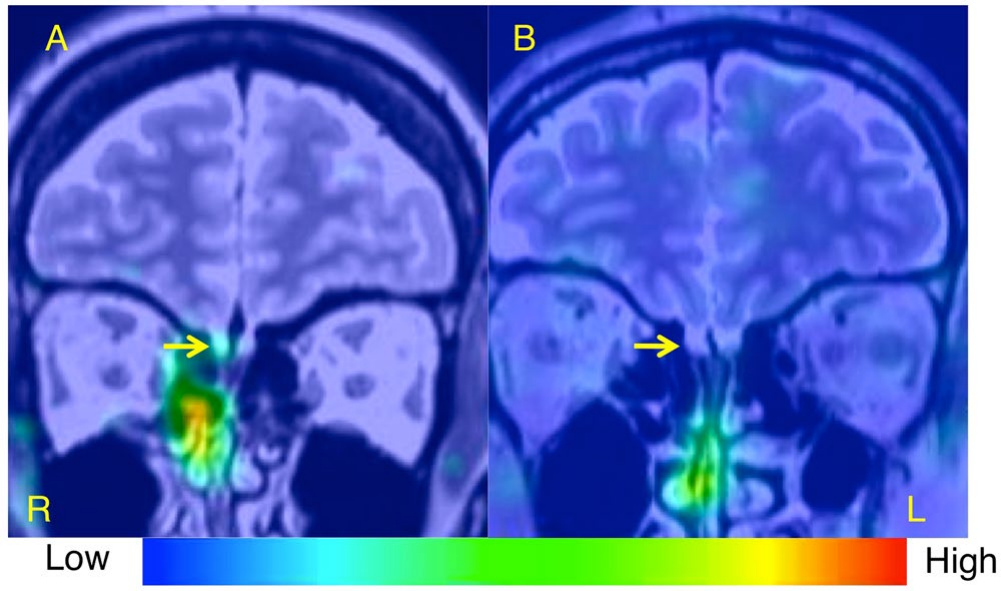
Single photon emission computed tomography (SPECT), X-ray computed tomography (CT), and magnetic resonance imaging (MRI) Olfacto-scintigraphy in representative cases with idiopathic olfactory dysfunction. (**A**) A case of better prognosis was a 49-year-old female who had relatively high nasal ^201^Tl migration to the olfactory bulb (11.1%). (**B**) A case of worse prognosis was a 40-year-old male who had low nasal ^201^Tl migration to the olfactory bulb (3.3%).

Below, we describe two representative cases of patients with idiopathic olfactory dysfunction. The case with the better prognosis was a 49-year-old female who had relatively high nasal ^201^Tl migration to the olfactory bulb (11.1%; Fig. 3A). The T&T odor recognition threshold decreased from 4.2 to 0.8 after treatment with the tokishakuyakusan for a month. The case with the worse prognosis was a 40-year-old male who had low nasal ^201^Tl migration to the olfactory bulb (3.3%; Fig. 3B). The T&T odor recognition threshold increased from 5.0 to 5.8 after treatment with the tokishakuyakusan for 16 months.

### Multivariable analysis of nasal ^201^Tl migration to the olfactory bulb, gender and age in patients

As olfaction may be correlated with gender and age, we conducted a multivariable analysis to determine whether nasal ^201^Tl migration to the olfactory bulb was the independent significant prognostic marker in the patients. Nasal ^201^Tl migration to the olfactory bulb was the significant independent predictors of prognosis in the patients with idiopathic olfactory dysfunction (Table 2). None of gender or age was significantly correlated with prognosis in the subjects (Table 2).

**Table 2:** Multivariable analysis of prognostic factors.

Prognostic factor	Risk ratio	P value
	(95% CI of Ratio)	(Proportional hazards regression approach)
Thallium-201 olfactory transport rate*		0.0001
<4.6% [low]	1.0	
≥4.6% [high]	5.3 × 10^9^ (7.0–2.9 × 10^30^)	
Gender		0.73
male	1.0	
female	0.7 (0.1–5.6)	
Age		0.48
≥60 years	1.0	
<60 years	2.3 (0.2–27.4)	

*Data dichotomized at the optimal cutoff value.

## Discussion

In this study, we showed that nasal ^201^Tl migration to the olfactory bulb determined before the conventional treatment with the Japanese herbal medicine tokishakuyakusan was a useful predictor of prognosis in idiopathic olfactory-impaired patients. Nasal ^201^Tl migration to the olfactory bulb has been shown to be significantly decreased by the transection of olfactory nerve fibers *in vivo* ^5^. Our results support the prognostic value of nasal ^201^Tl migration to the olfactory bulb, suggesting that patients with intact olfactory nerve fibers could be selected using thallium-based imaging for the long-term follow-up of olfactory dysfunction.

Intravenous injection of thiamine propyldisulfide (Alinamin) induces the sensation of a garlic-like odor, and Alinamin injection is widely used as one of the subjective olfactory tests in Japan^13^. Nonresponders in the Alinamin test have been previously shown to have poor prognosis in the recovery of olfactory acuity^14^. The Alinamin test is simple but often results in vascular pain; the Alinamin test was not assessed here because five patients refused to undertake the test because of the associated pain.

The olfactory bulb volume has been shown to be a predictor of olfactory recovery in patients with postinfectious and posttraumatic olfactory loss^3^. In the present study, the olfactory bulb volume was not significantly correlated with the duration until recovery of olfactory recognition, although the data for olfactory bulb volume were dichotomized according to a previous study (<40 mm^3^ vs. ≥40 mm^3^)^3^. The reason for this discrepancy may be that the subjects in this study, but not those in the previous study, suffered from idiopathic olfactory disorder.

To evaluate olfactory nerve connectivity in patients with impaired olfaction, assessing the migration of nasally administered ^201^Tl to the olfactory bulb is a useful and safe method. None of the subjects examined here experienced major side effects following the nasal administration of ^201^Tl, perhaps due to the small dose of administered ^201^Tl. The absorbed doses of ^201^Tl were estimated as 0.59 mGy in the lens and 0.067 mGy in the brain after the nasal administration of 22 MBq ^201^Tl by applying the MIRD formula^7, 15^. Thus far, no serious side effects have been reported in subjects after olfacto-scintigraphy. Furthermore, because the facial bone surrounds the nasal turbinates, acute radiation effects to the neighboring organs, such as the lens and brain, may be tolerable.

Because olfacto-scintigraphy takes 24 h to assess ^201^Tl migration to the olfactory bulb, patients have to return to the hospital for SPECT-CT analysis 24 h after ^201^Tl administration. Therefore, for clinical applications, there is a need for an olfactory nerve tracer that can rapidly enter the central nervous system. Iodine-125-conjugated human recombinant insulin-like growth factor-1 (^125^I-hIGF-1) uptake can be detected in the mouse cerebrum 30 min after nasal administration^16^. Furthermore, unilateral olfactory bulb resection prevents nasally administered hIGF-1 from increasing the phosphorylation of extracellular signal-regulated kinase 1/2 in the mouse cerebrum *in vivo* ^16^. These findings suggest that olfactory bulb damage reduces the nasal transport of hIGF-1 to the brain *in vivo*. Although ^125^I-hIGF-1 is commercially available, its half-life (60.1 days) is too long for clinical applications. Furthermore, the low energy of Iodine-125 is not appropriate for SPECT imaging. Thus, the development of a new isotope-conjugated hIGF-1 for nasal administration warrants further investigation.

In this study, patients with idiopathic olfactory dysfunction received conventional treatment through the oral intake of the Japanese herbal medicine tokishakuyakusan. In contrast, the effect of olfactory training using four intense odors (rose, eucalyptus, lemon and cloves) has been reported in the treatment of patients with olfactory loss in Germany^17^. Olfactory training is not a conventional treatment for patients with olfactory loss in Japan because it is not clear which odors are useful for the olfactory training of Japanese patients. Thus, further investigation is required to determine whether nasal ^201^Tl migration to the olfactory bulb may serve as a predictor of prognosis following the olfactory training of patients with olfactory disorders.

In conclusion, the duration until recovery of odor recognition was significantly shorter in patients with idiopathic olfactory dysfunction with high nasal ^201^Tl migration to the olfactory bulb than in those with low nasal ^201^Tl migration to the olfactory bulb in this retrospective study. Our results suggest that severe olfactory nerve damage determined using thallium-based olfactory imaging predicts a worse prognosis in patients with idiopathic olfactory loss. None of the other potential prognostic factors tested (gender, age, T&T odor recognition threshold, smoking habit, parosmia, or olfactory bulb volume) was significantly correlated with prognosis in the subjects. Our thallium-based imaging “Olfacto-scintigraphy” is useful for avoiding the ineffective treatment in the patients, although the subjects need to return to hospital 24 h after nasal administration of ^201^Tl. To assess whether nasal ^201^Tl migration to the olfactory bulb is a general predictor of prognosis in patients with olfactory disorders, this study needs to be extended to a larger cohort across multiple institutes following the olfactory training of patients with olfactory impairment.

## Materials and Methods

### Subjects

Twenty-four subjects with idiopathic olfactory disorders were randomly selected from among patients who received follow-up visits at the smell clinic at the Kanazawa Medical University Hospital (2009–2016) and who underwent thallium-based olfactory imaging (olfacto-scintigraphy) (7 women and 17 men; aged 23–71 years). Subjects were excluded if they had a history of chronic rhinosinusitis, head injury, acute respiratory infection in the three months prior to the onset of olfactory dysfunction, congenital olfactory loss, or neurodegenerative disorder. The Medical Ethics Committees of Kanazawa Medical University and Kanazawa University approved this study in advance (trial registration number: UMIN000023519). All subjects were informed of the objectives of the study and possible side effects (e.g., allergic reactions to ^201^TlCl and irritation of the digestive system) and gave written informed consent. Subjects were excluded if they were pregnant or lactating or had a history of kidney disease, liver injury, or other serious illness.

The characteristics of the patients are as follows (T&T odor recognition threshold, 4.8 ± 1.0 [mean ± SD]; Smoking habit, 7 smokers/ex-smokers and 17 non-smokers; Parosmia, 6 patients with parosmia and 18 patients without parosmia).

In this study, the subjects with olfactory dysfunction were observed the day after olfacto-scintigraphy until the “recovery” of olfactory recognition or the end of the observation time. The patients with idiopathic olfactory dysfunction received conventional treatment via the oral intake of tokishakuyakusan (TJ-23, Tsumura, Tokyo, Japan) at the standard dose of 7.5 mg per day at our institute.

The duration until the “recovery” of olfactory recognition or the end of the observation time ranged from 1 to 60 months (11 ± 12 months [mean ± SD]: see definition of “recovery” as follows). The improvement in the T&T odor recognition threshold was judged according to the criteria of the Japanese Rhinologic Society^12^ (cure, T&T odor recognition threshold ≤2.0; remission, decrease in T&T odor recognition threshold ≥1.0; progress, increase in T&T odor recognition threshold of ≥1.0; stable, all other cases). Cure or remission was defined as “recovery”^12^.

### T&T olfactometry

In Japan, T&T olfactometry (Daiichi Yakuhin Sangyo, Tokyo, Japan) is a standard means of measuring olfactory thresholds; the normal odor recognition threshold score of each nostril is 2.0 or less. The olfactory severities were categorized according to the mean T&T recognition thresholds, and patients were diagnosed as anosmia when the T&T recognition thresholds were 5.6 or greater^12^.

Each odorant was dissolved in mineral oil to form a graded series of concentrations and then was applied liberally to blotting paper, followed by presentation to patients using T&T olfactometry. The odor recognition threshold was measured on the nasal side of ^201^Tl administration. The T&T odor recognition thresholds in the subjects ranged from 2.6 to 5.8 (4.8 ± 1.0 [mean ± SD]).

### Olfacto-scintigraphy and analysis of olfactory bulb volume

For each subject, 0.3 ml of ^201^TlCl (22 MBq) was administered unilaterally to the olfactory cleft, and SPECT-CT was conducted 24 h later. The data acquisition was performed using a dual-headed SPECT-CT hybrid system (Symbia T6; SIEMENS Healthcare Japan, Tokyo, Japan) equipped with low-energy high-resolution collimators. The data were acquired over 360°, with 90 projections (30 s per projection), 128 × 128 matrix (with 2.29 times zoom, pixel size 2.1 mm), and a 72-KeV photopeak with a 30% main window and 7% subwindow on both sides. Data reconstruction was performed with 3D OSEM (nine subsets and eight iterations), including attenuation and scatter correction (Flash 3D, SIEMENS).

Olfactory clefts were open in all patients. Separate MRI images were merged with the SPECT-CT images. Experienced radiologists (NW and HT) who were blind to the olfactory test data separately determined the volume of the olfactory bulb by manual segmentation of 3.0 Tesla MRI coronal slices (2-mm thick, T2 weighted image) of the olfactory bulb on the nasal side of ^201^Tl administration. The change in the diameter at the beginning of the olfactory tract was used as the proximal demarcation of the olfactory bulb. The averages of the two radiologists’ scores were used for the statistical analysis.

The index of ^201^Tl migration from the olfactory epithelium to the olfactory bulb was determined as the ratio of the total ^201^Tl count in the olfactory bulb region of interest to the total ^201^Tl count in the nasal turbinate region of interest, expressed as a mean percentage of the values calculated from both sagittal and coronal images. Experienced nuclear radiologists (JT, KO, and SK) who were blind to the olfactory test data determined two separate regions of interest for the nasal turbinate area and the anterior skull base (olfactory bulb area) on the ^201^Tl SPECT–MRI fusion image. On 3 sequential fused images in both sagittal and coronal planes, large regions of interest were tentatively set manually on the nasal turbinate area to cover all of the residual ^201^Tl activity in the nasal cavity, and the nasal cavity region of interest was defined as the area bounded by the 50% threshold of the peak ^201^Tl count. Next, the oval regions of interest were set manually to delineate the olfactory bulb on the side of the nasal administration of the tracer by referencing the MRI T2-weighted images.

### Definition of recovery and analysis of prognostic factors

We assessed whether the duration until recovery correlated with prognostic factors, including nasal ^201^Tl migration to the olfactory bulb (<4.6% [low] vs. ≥4.6% [high]; data dichotomized at the optimal cutoff value [Table 3]), gender (male vs. female), age (≥60 years. vs. <60 years), T&T odor recognition thresholds (≥5.6 [anosmia] vs. <5.6 [dysosmia]), smoking habit (smokers and ex-smokers vs. non-smokers), parosmia (present vs. absent) and olfactory bulb volume (<40 mm^3^ vs. ≥40 mm^3^; data dichotomized according to the previous study^3^). We did not assess whether disease duration correlated with the prognosis of the patients because some patients did not clearly remember when they started to have olfactory dysfunction.

**Table 3:** Sensitivity and specificity at the cutoff values of nasal ^201^Tl migration to the olfactory bulb between patient groups with and without recovery at 6 months after olfacto-scintigraphy.

Cutoff value	Sensitivity (%)	Specificity (%)
(Nasal ^201^Tl migration to the olfactory bulb (%))		
11.1	100	20
9.1	100	40
8.2	95.0	40
5.8	95.0	60
5.6	95.0	80
4.8	89.5	80
4.6*	89.5	100
4.2	84.2	100
3.7	79.0	100
3.5	68.4	100
3.3	63.2	100

*Optimal cutoff value.

### Statistical analysis

For statistical analysis, log-rank tests, two-tailed Spearman correlations, and Mann–Whitney tests were performed using Prism 5 software (GraphPad, San Diego, CA, USA). Multivariable statistical models were generated using a proportional hazards regression approach (JMP 13 software, Cary, NC, USA). The sensitivities and specificities in the receiver operating characteristic (ROC) table were also calculated using JMP 13 software (Cary, NC, USA). P values less than 0.05 were considered significant.

### Compliance with ethical standards

All of the procedures performed in this study involving subjects were in accordance with the ethical standards of the institutional committee and with the 1964 Helsinki Declaration and its later amendments or comparable ethical standards. Informed consent was obtained from all of the individual participants included in the study. This article does not contain any studies with animals performed by any of the authors.
